# A novel strategy for spinal cord reconstruction via vascularized allogeneic spinal cord transplantation combine spinal cord fusion

**DOI:** 10.1111/cns.70020

**Published:** 2024-09-23

**Authors:** Weihua Zhang, Rongyu Lan, Tingting Shen, Jie Qin, Zhihui Wang, Jiayang Chen, Jiaxing Wang, Zhuotan Wu, Yudong Xu, Yangyang Shen, Qikai Lin, Yuan Chen, Yi Wei, Yiwen Liu, Yuance Ning, Yiyan Zhou, Liji Deng, Linxuan Han, Xiaofei Wu, Haixuan Deng, Zhenbin Cao, Xianping Yao, Xiaoping Ren

**Affiliations:** ^1^ Department of Orthopedics Ruikang Hospital Affiliated to Guangxi University of Chinese Medicine Nanning Guangxi China; ^2^ Institute of Orthopedics, Ruikang Hospital Affiliated to Guangxi University of Chinese Medicine Nanning Guangxi China; ^3^ Global Initiative to Cure Paralysis (GICUP Alliance) Columbus Ohio USA; ^4^ Guangxi University of Chinese Medicine Nanning Guangxi China; ^5^ Department of Medicine School Guangxi University Nanning Guangxi China; ^6^ Department of Anatomy and Cell Biology McGill University Montreal Quebec Canada; ^7^ Department of Pharmacology and Toxicology University of Toronto Toronto Ontario Canada; ^8^ Department of Imaging Ruikang Hospital Affiliated to Guangxi University of Chinese Medicine Nanning Guangxi China; ^9^ Hangzhou Research Institute of Chemical Industry Hangzhou Zhejiang China

**Keywords:** polyethylene glycol, spinal cord defect, spinal cord fusion, spinal cord injury, tacrolimus, vascularized allogeneic spinal cord transplantation

## Abstract

**Aims:**

Spinal cord injuries (SCI) pose persistent challenges in clinical practice due to the secondary injury. Drawing from our experience in spinal cord fusion (SCF), we propose vascularized allogeneic spinal cord transplantation (vASCT) as a novel approach for SCI, much like organ transplantation has revolutionized organ failure treatment and vascularized composite‐tissue allotransplantation has addressed limb defects.

**Materials and Methods:**

In this study, 24 dogs were paired and underwent vASCT, with donor spinal cord grafts and polyethylene glycol (PEG) application for SCF. The experimental group (*n* = 8) received tacrolimus and methylprednisolone, while the control group (*n* = 4) received only methylprednisolone. Safety and efficacy of vASCT were evaluated through electrophysiology, imaging, and 6‐month follow‐up.

**Results:**

The experimental group showed substantial recovery in hind limb motor function. Imaging revealed robust survival of spinal cord grafts and restoration of spinal cord continuity. In contrast, the control group maintained hind limb paralysis, with imaging confirming spinal cord graft necrosis and extensive defects. Electrophysiologically, the experimental group exhibited restored motor evoked potential signal conduction postoperatively, unlike the control group. Notably, PEG application during vASCT led to signal conduction recovery in intraoperative spinal cord evoked potential examinations for all dogs.

**Conclusion:**

In the vASCT surgical model, the combination of PEG with tacrolimus has demonstrated the ability to reconstruct spinal cord continuity and restore hind limb motor function in beagles. Notably, a low dose of tacrolimus has also exhibited an excellent anti‐immune rejection effect. These findings highlight vASCT's potential promise as a therapeutic strategy for addressing irreversible SCI.

## Graphical abstract

In this work, we proposed that vascularized allogeneic spinal cord transplantation (vASCT) might offer a new surgical approach for treating spinal cord injury. In the vASCT surgical model, the combination of polyethylene glycol with tacrolimus demonstrated the ability to reconstruct spinal cord continuity and restore hind limb motor function in beagles. The safety and efficacy of vASCT were verified by behavioral evaluation, electrophysiological examination and imaging examination.

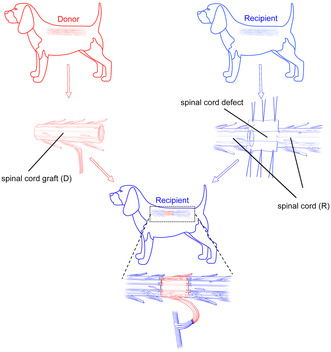

## 1 INTRODUCTION

In the realm of spinal cord injury (SCI), the challenge of effectively treating paraplegia resulting from such injuries remains unmet in clinical practice. Our prior research has specifically highlighted the capacity of polyethylene glycol (PEG) to repair axonal membranes in various animal models, including mice, rats, beagles, and monkeys, all of which underwent spinal cord transection.^1^, ^2^, ^3^, ^4^, ^5^ In order to translate these promising results from animal studies into clinical applications, we have devised several spinal cord fusion (SCF) techniques, such as vascular pedicle hemisected spinal cord transplantation (vSCT)^6^, ^7^ and sural nerve transplantation (SNT).^8^ The findings from pertinent preclinical experiments and clinical trials have demonstrated the potential of SCF to restore spinal cord neural continuity and partially recover neurological function below the level of the SCI.^6^, ^7^, ^8^ However, our exploration of these clinical trials has revealed certain limitations in the current approaches to SCF. Patients with paraplegia caused by a SCI located close to the cauda equina nerve are not appropriate candidates for vSCT; for them, SNT becomes the sole viable option. It's important to recognize that SNT involves grafting nerve tissue from the patient's lower extremity, which differs somewhat from spinal cord tissue in terms of structure and the number of fibers. Consequently, this variance presents certain constraints on the extent of postoperative functional recovery. In light of these observations, we contend that there is a need for optimization in the treatment of spinal cord injuries.

The field of organ transplantation has witnessed substantial advancements. Organ transplantation has evolved into a pivotal therapeutic approach for addressing severe organ failure on a global scale.^9^ A notable stride beyond traditional organ transplantation is the emergence of vascularized composite‐tissue allotransplantation (vCTA). The vCTA comprises multiple tissue types transplanted collectively as a single unit, exemplified by intricate procedures such as hand transplants, which encompass muscle, skin, bone, blood vessels, and nerves.^10^, ^11^ In recent years, the vCTA has gained considerable prominence in clinical practice, particularly for patients with injuries that are beyond the scope of conventional plastic surgery for repair, and it raises an intriguing question: Can the vCTA hold potential as a treatment approach for traumatic spinal cord injuries characterized by significant spinal cord tissue loss?

There have been reports from other research groups highlighting the effectiveness of partially restoring hind limb motor function through the allotransplantation of segments of adult spinal cord tissues into spinal cord defects in rodent and canine animal models.^12^, ^13^ However, our team posited that a mature allogeneic spinal cord transplantation should adhere to the fundamental principles of organ transplantation and vCTA. In this context, the donated spinal cord tissue should incorporate a vascular pedicle, facilitating the reestablishment of blood circulation.

**FIGURE 1:**
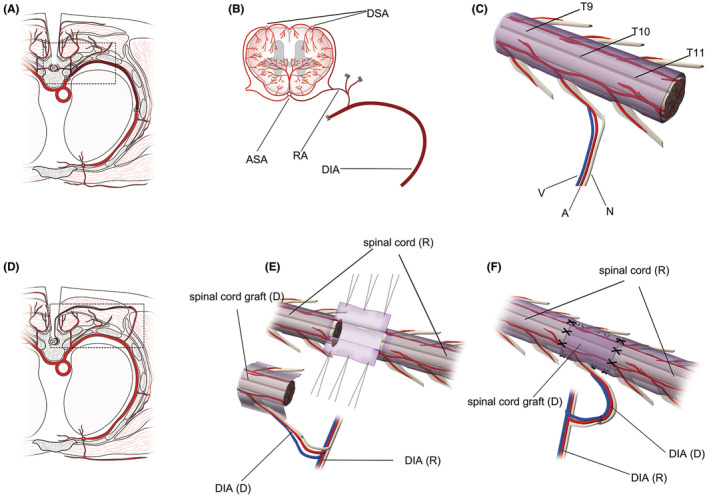
Key surgical procedures and anatomy of vASCT. Cross‐sectional illustration of the T10 level of the donor before isolating the graft (A). Cross‐sectional view and 3D imaging depicting the spinal cord graft from the donor (B, C). Donor spinal cord tissue harvested from T9–T11 levels (C), along with the radicular artery (RA), dorsal intercostal artery (DIA) and accompanying vein at the T10 level serving as its vascular pedicle (B, C). Cross‐sectional representation of T10 levels in recipients after vascular anastomosis and spinal cord transplantation (D). 3D imaging illustrating vascular anastomosis and spinal cord transplantation (E, F). The vascular pedicle of the donor spinal cord graft was anastomosed end‐to‐end with the muscular vascular perforator of the DIA at the T10 level of the recipient. The recipient spinal cord was exposed by an “H”‐shaped incision in the dura mater at the T10 level, and a segment of spinal cord tissue was excised to create a spinal cord defect. The length of the donor spinal cord graft was tailored appropriately to match the recipient spinal cord defect, with the dura cut longitudinally ventral to the graft (E). Finally, the graft was transplanted to bridge the recipient spinal cord stumps, and the dura mater of both donor and recipient was sutured to prevent cerebrospinal fluid leakage (F). A, artery; ASA, anterior spinal artery; D, donor; DSA, dorsal spinal artery; N, nerve; R, recipient; V, vein.

**FIGURE 2:**
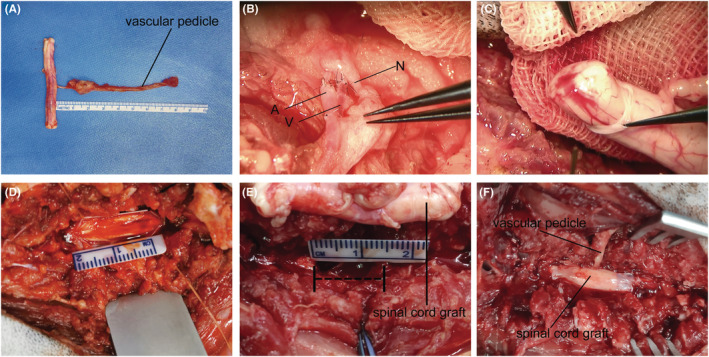
Intraoperative operating photographs of vASCT. Sequential intraoperative images of the vASCT procedure are depicted. Firstly, the vascularized spinal cord tissue was separated from the donor (A). Subsequently, the vascular pedicle of the donor spinal cord graft was anastomosed with the muscular vessel perforator of the dorsal intercostal artery (DIA) at the T10 level of the recipient (B). Following the completion of vascular anastomosis, careful examination of the blood exudation from the stumps of the donor spinal cord graft was conducted to verify the successful reconstruction of its blood supply (C). The dura mater of the recipient spinal cord was then incised (D), and a 1.5 cm segment of spinal cord tissue was excised to create a spinal cord defect (E). Finally, an appropriately sized spinal cord graft was tailored to span the stumps of the recipient spinal cord, and the dura mater was meticulously sutured and closed (F).

In our research, we have introduced a novel approach to allogeneic spinal cord transplantation in beagles. The spinal cord graft from our donor animals included the radicular artery (RA)‐dorsal intercostal artery (DIA), along with an accompanying vein. In the recipient animals, this vascular pedicle of the spinal cord graft was meticulously joined with the muscle perforating branches of the DIA, along with its accompanying vein, at the T10 level of the donor. This intricate vascular connection was established to ensure a robust blood supply to the spinal cord graft (Figures 1 and 2). Our investigation yielded compelling results through behavioral assessments, electrophysiological examinations, and imaging studies, collectively confirming the safety and effectiveness of vASCT in beagles presenting with acute spinal cord defects.

## 2 MATERIALS AND METHODS

### 2.1 Animals

In this study, a total of 24 female beagles, weighing between 8 and 10 kg, were utilized (Table 1). To ensure equitable and impartial allocation, these 24 beagles were randomly paired, resulting in 12 pairs (one donor paired with one recipient). Subsequently, these 12 pairs were randomly assigned to two groups: an experimental group comprising eight pairs and a control group comprising four pairs. The recipient beagles in the experimental group underwent a treatment protocol involving the oral administration of tacrolimus (Hangzhou Sino‐American East China Pharmaceutical Co., LTD, Hangzhou, China) at a dosage of 0.1 mg/kg/day, coupled with intramuscular injections of methylprednisolone (Liaoning HAISCO Pharmaceutical Co. LTD, Xingcheng, China) at a dosage of 1.0 mg/kg/day post‐surgery to mitigate immune rejection. In contrast, recipient beagles in the control group received intramuscular injections of methylprednisolone at the same dosage after surgery to address immune rejection. Furthermore, it is imperative to emphasize that all animal‐related procedures were rigorously reviewed and approved by the Institutional Animal Care and Use Committee of the Guangxi University of Chinese Medicine (DW20230525‐089). These procedures were carried out in strict accordance with Directive 2010/63/EU of the European Parliament. Moreover, the reporting of animal data adhered to The ARRIVE 2.0 guidelines.^14^ The beagles utilized in this study were sourced from Guangzhou General Institute of Medical Research Co. LTD and were provided with comfortable living conditions, including a 12‐h light and dark cycle. Additionally, they had access to food and water ad libitum.

**TABLE 1:** General conditions of beagles in experimental and control groups.

	Experimental group								Mean ± SD	Control group				Mean ± SD	*t* Value	*p* Value
	E1	E2	E3	E4	E5	E6	E7	E8		C1	C2	C3	C4			
Sex	Female	Female	Female	Female	Female	Female	Female	Female	–	Female	Female	Female	Female	–	–	–
Weigh (kg)	8.2	8.3	9.1	8.5	8.7	8.1	9.2	8.0	8.51 ± 0.45	8.0	8.5	9.3	8.8	8.65 ± 0.54	−0.466	0.651
Time of surgery (min)	300	250	300	270	175	170	180	185	228.75 ± 57.24	260	310	178	205	238.25 ± 58.76	−0.269	0.794
Time of ischemia (min)	132	160	172	145	76	85	100	90	120.00 ± 32.91	155	115	84	170	131.00 ± 39.00	−0.478	0.643
Postoperative cerebrospinal fluid leakage	No	No	No	No	No	No	No	No	–	No	No	No	No	–	–	–
Postoperative pneumonia	No	No	Yes	No	No	No	No	No	–	No	No	No	No	–	–	–
Postoperative pressure sore	No	No	No	No	No	No	No	No	–	Yes	Yes	Yes	Yes	–	–	–
Postoperative urinary tract infection	No	No	No	No	No	No	No	No	–	No	No	No	No	–	–	–
Postoperative bowel and urine function	No	No	No	No	No	No	No	No	**–**	No	No	No	No	–	–	–

Abbreviation: SD, standard deviation.

### 2.2 Procedure performed on the donor

The surgical procedure involved making incisions through the layers of muscles, fat, and skin above the thoracic spine, specifically at the T9–T11 levels. This allowed for the performance of laminectomy to expose the dura mater. For the purposes of this study, we selected the RA‐DIA and its associated vein on one side at the T10 level to serve as the vascular pedicle for the spinal cord tissue (Figure 1A,B). Under microscopic guidance, meticulous isolation of the vascular‐pedicle‐bound spinal cord tissue (T9–T11) was performed using specialized microinstruments, ensuring the preservation of both the spinal cord and its vascular pedicle (Figures 1C and 2A). The extracted spinal cord tissue was carefully enveloped in ice‐cold saline‐soaked gauze and maintained at a low temperature. Subsequently, the donor animal was humanely euthanized in compliance with ethical and scientific standards.

### 2.3 Procedure performed on the recipient

As one group of surgeons operated on the donor beagles, a parallel group simultaneously performed surgical procedures on the recipient beagles. Longitudinal incisions were made in the skin and subcutaneous soft tissue at the T9–T11 levels, allowing for the exploration and exposure of the muscle perforators of the DIA at the T10 level within the paravertebral region of the recipient, on the same side as the donor spinal cord graft vascular pedicle. The vascular pedicle of the donor spinal cord graft was meticulously anastomosed with the muscle perforator and the accompanying vein of the recipient DIA (Figures 1D,E and 2B). After assessing the blood circulation of the donor spinal cord graft (Figure 2C), laminectomy was performed to expose the T10 segment, as well as the lower edge of T9 and the upper edge of T11 of the recipient spinal cord (Figure 2D). A “H” shape incision was made in the dura mater, and a segment of spinal cord tissue at the T10 level was excised with an extremely sharp knife to create a 1.5 cm spinal cord defect (Figures 1E and 2E). The length of the donor spinal cord graft was appropriately tailored to match the extent of the spinal cord defect, and it was meticulously bridged at both the distal and proximal stumps of the recipient spinal cord. A 2 mL of PEG‐600 (100%, Sigma‐Aldrich/Merck) was applied topically to the two contact interfaces of spinal cord after the transplantation. Finally, the dura mater was sutured (Figures 1F and 2F), and the incision was meticulously closed through a layer‐by‐layer suturing process.

### 2.4 Postoperative care

All beagles participating in the study received intramuscular injections of 25 mg/kg/day of ceftriaxone sodium (Shanghai Roche Pharmaceutical Co., LTD, Shanghai, China) for a duration of 7 days following the surgical procedures. In addition, intravenous administration of 100 IU/kg/day of heparin (Maanshan Fengyuan Pharmaceutical Co., LTD, Maanshan, China) was provided to the beagles undergoing vASCT for 7 days postoperatively. The beagles were assisted in defecation and urination through abdominal massage three times a day throughout the observation period of the study, or until the restoration of the voiding reflex.

### 2.5 Behavioral observation

Two trained examiners conducted comprehensive assessments on all the beagles to monitor their recovery of hind limb function. The assessments employed the canine Basso‐Beattie‐Bresnahan (cBBB) rating scale. This rating scale assigns a cBBB score ranging from 0, representing complete paraplegia, to 19, indicating normal functionality.^15^ Each individual observation session was conducted for a minimum duration of 10 min to ensure a thorough and accurate evaluation.

### 2.6 Electrophysiological examination

The electrophysiological examination in this study encompassed two distinct phases: intraoperative electrophysiological assessment and postoperative electrophysiological evaluation. The intraoperative examination focused on spinal cord evoked potential (SCEP) examinations. To evaluate the electrophysiological conductivity of the spinal cord, three SCEP examinations were conducted at three critical time points: before the induction of the spinal cord defect, after the spinal cord defect was induced, and at 15 min after the transplantation and PEG topically applied. Following the exposure of the spinal cord by opening the dura mater, SCEP was carried out by positioning specialized electrodes for both stimulation and recording (REP‐1.5Z, Suzhou Lepps Electronics Co., Ltd, China) on the dorsal surface of the spinal cord. The stimulation electrode administered a constant voltage stimulation of 1 V. Regular pulses of constant stimulation current (17 Hz, 0.025 ms duration) were consistently delivered. Typically, an average of 30 consecutively recorded signals was used for each tracing. The NIM‐ECLIPSE® System (Medtronic, United States) was employed to record the signals with precision and reliability.

Two months following the surgery, motor evoked potentials (MEPs) were performed to complete the postoperative electrophysiological evaluation. MEPs were induced using brief transcranial electrical pulses with a pulse width of 75 μs and a high voltage of 150 V in an anodal electrical stimulus train. The stimulating electrodes were inserted over the motor cortex regions. Meanwhile, the recording electrodes were strategically placed sequentially at the abductor pollicis longus (APL) of the forelimbs and the biceps femoris (BF), tibialis anterior (TA), and flexor hallucis longus (FHL) of the hindlimbs.

### 2.7 Imaging examination

All animals underwent Magnetic Resonance Imaging (MRI) and Diffusion Tensor Imaging (DTI)^16^ using a 1.5T MRI system (Achieva 3, Philips, Amsterdam, The Netherlands). DTI is an advanced MRI technique that allows for the assessment of the microstructural integrity of nerve fiber tracts.^17^ The imaging procedures were conducted with the animals in a prone position while under anesthesia. These imaging assessments included sagittal T2‐weighted Fast Spin‐Echo sequences (TR = 1300 ms; TE = 115 ms; slice thickness = 4 mm; slice gap = 0.6; NSA = 2) and axial single‐shot Echo‐planar DTI sequences (TR = 2782 ms; TE = 83 ms; voxel size = 2.34 mm × 2.46 mm; slice thickness = 5 mm; slice gap = 0.06; NSA = 4; diffusion directional resolution = 32). These imaging sequences were conducted 2 months following the surgical procedures.

### 2.8 Statistical analysis

In the current study, we conducted data analysis using SPSS 20.0 (IBM Inc. SPSS Statistics, Armonk, NY, USA) for data processing and statistical analysis and GraphPad Prism 9 (GraphPad Software, La Jolla, CA, USA) for generating graphical representations. We employed unpaired *t*‐tests for comparative analysis. The presentation of data is conveyed as the mean ± standard deviation (SD), with statistical significance set at *p* < 0.05. This approach ensures a rigorous and scientifically sound methodology for our analysis and presentation of results.

## 3 RESULTS

### 3.1 Behavioral recovery after vASCT

**FIGURE 3:**
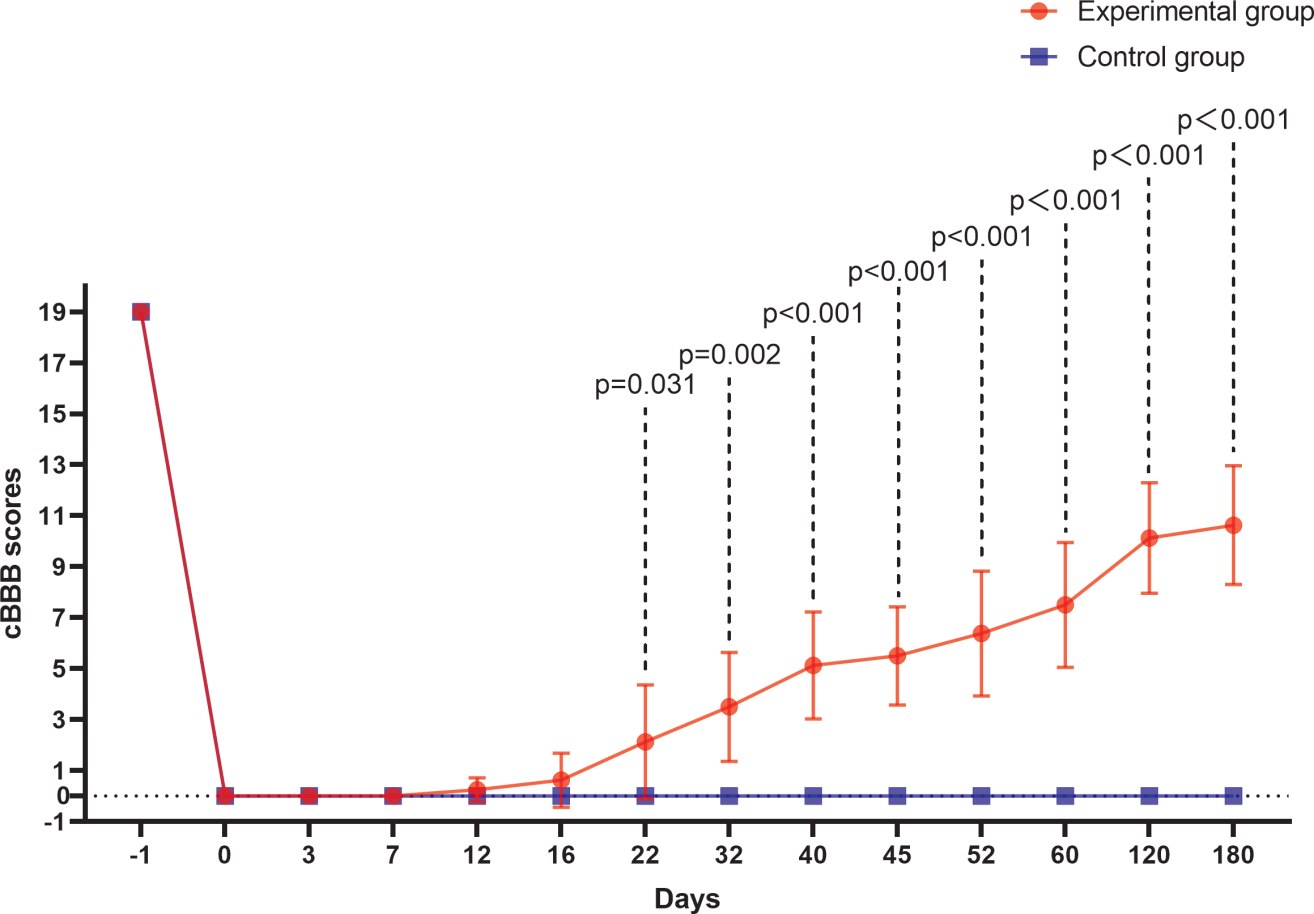
Comparison of hind limb motor function recovery. The recovery of hind limb motor function was evaluated in both the experimental and control groups 6 months post‐surgery. In the experimental group, certain beagles exhibited spontaneous hind limb movement as early as 12 days post‐surgery, with continuous improvement observed over the subsequent 6 months. In contrast, the control group remained paralyzed for the entire 6‐month postoperative period, registering a cBBB score of 0. A significant disparity (*p* < 0.05) in cBBB scores between the experimental and control groups was noted from day 22 post‐surgery.

From the first day following vASCT, we assessed the recovery of hind limb motor function in both the experimental and control groups using the cBBB score. In the experimental group, two beagles exhibited voluntary movement in the hind limb joints (scoring a one) 12 days post‐surgery. At the 6‐month mark, two beagles demonstrated frequent plantar stepping and consistent forelimbs‐hindlimbs coordination, scoring a 14 (Video S1). The average cBBB score for beagles in the experimental group was 10.63 at the 6‐month evaluation. Conversely, in the control group, there was no observed recovery in hind limb motor function. The hind limb remained paralyzed with a cBBB score of 0, and there was an abnormal increase in muscle tone 6 months after the surgery (Video S1). We conducted an unpaired *t*‐test to analyze the behavioral cBBB scores of the two groups of beagles. The results indicated that there was a statistically significant difference between the experimental group and the control group from 22 days to 6 months post‐surgery (*p* < 0.05, Figure 3, Table 2). These findings underscore the notable distinctions in motor function recovery between the two groups.

**TABLE 2:** Behavioral recovery of beagles in experimental and control groups for 6 months.

cBBB scores																
Days	Experimental group								Mean ± SD	Control group				Mean ± SD	*t* Value	*p* Value
	E1	E2	E3	E4	E5	E6	E7	E8		C1	C2	C3	C4			
−1	19	19	19	19	19	19	19	19	19 ± 0	19	19	19	19	19 ± 0	–	–
0	0	0	0	0	0	0	0	0	0 ± 0	0	0	0	0	0 ± 0	–	–
3	0	0	0	0	0	0	0	0	0 ± 0	0	0	0	0	0 ± 0	–	–
7	0	0	0	0	0	0	0	0	0 ± 0	0	0	0	0	0 ± 0	–	–
12	0	0	1	0	0	1	0	0	0.25 ± 0.46	0	0	0	0	0 ± 0	1.528	0.170
16	0	0	1	0	3	1	0	0	0.63 ± 1.06	0	0	0	0	0 ± 0	1.667	0.140
22	1	1	1	3	7	3	1	0	2.13 ± 2.23	0	0	0	0	0 ± 0	2.693	0.031
32	3	3	4	3	7	6	1	1	3.50 ± 2.14	0	0	0	0	0 ± 0	4.630	0.002
40	3	4	7	3	8	7	3	6	5.13 ± 2.10	0	0	0	0	0 ± 0	6.902	<0.001
45	3	4	7	3	8	7	6	6	5.50 ± 1.93	0	0	0	0	0 ± 0	8.072	<0.001
52	3	7	10	3	8	8	6	6	6.38 ± 2.45	0	0	0	0	0 ± 0	7.372	<0.001
60	3	7	11	7	8	8	6	10	7.50 ± 2.45	0	0	0	0	0 ± 0	5.976	<0.001
120	7	10	14	12	10	8	10	10	10.13 ± 2.17	0	0	0	0	0 ± 0	9.119	<0.001
180	7	10	14	14	10	10	10	10	10.63 ± 2.33	0	0	0	0	0 ± 0	12.920	<0.001

Abbreviations: cBBB, canine Basso‐Beattie‐Bresnahan; SD, standard deviation.

### 3.2 Recovery of electrophysiological signal transduction during and after vASCT

**FIGURE 4:**
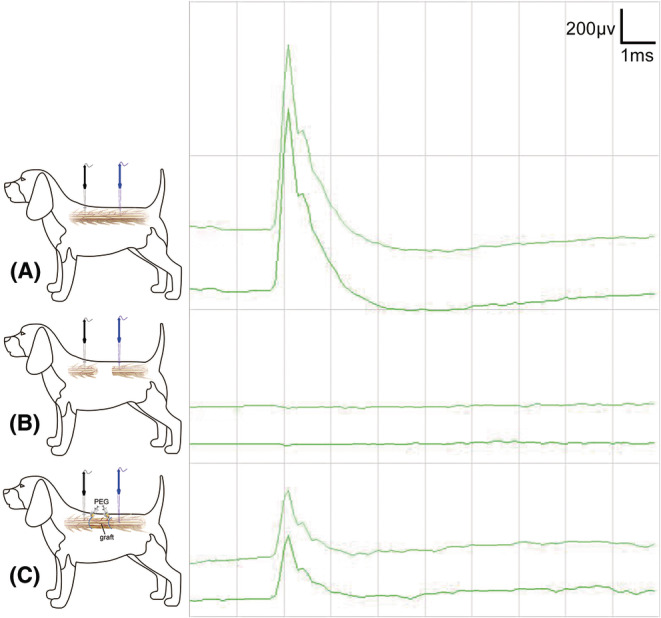
SCEP examination during vASCT. The SCEP examination was conducted in the course of vASCT. Initially, SCEP was performed prior to inducing the spinal cord defect, revealing normal waveforms (A). Subsequently, SCEP was repeated after the creation of the spinal cord defect, resulting in the disappearance of the positive waveform (B). Finally, the conclusive SCEP examination was carried out post‐spinal cord transplantation and topical application of PEG at the interfaces of the two spinal cord surfaces. A positive waveform emerged, albeit with a lower amplitude compared to the SCEP waveform recorded before the induction of the spinal cord defect (C). The stimulating electrode (black) was positioned on the proximal spinal cord surface, while the recording electrode (blue) was placed on the distal spinal cord surface.

SCEP were systematically assessed in all beagles undergoing vASCT. Prior to the spinal cord defect, all beagles exhibited normal SCEP waveforms and had intact spinal cord signal conduction capabilities (Figure 4A). Following the creation of the spinal cord defect, SCEP waveforms disappeared in all beagles (Figure 4B). Following spinal cord transplantation and the local application of PEG, the SCEP waveforms in both the experimental and control groups partially recovered (Figure 4C).

**FIGURE 5:**
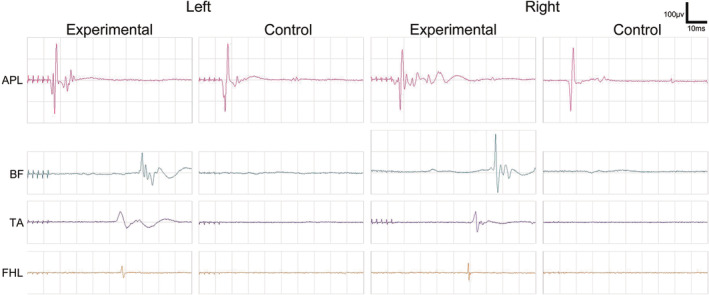
MEP Examination 2 months post‐surgery. Two months after surgery, positive MEP waveforms were recorded in the abductor pollicis longus (APL) of both forelimbs for both the experimental and control groups of Beagle dogs. Additionally, positive MEP waveforms were observed in the biceps femoris (BF), tibialis anterior (TA), and flexor hallucis longus (FHL) of both hind limbs in the experimental group. Notably, when compared with the APL of both forelimbs, these hind limb MEPs exhibited lower amplitudes and prolonged latencies. Conversely, in the control group, no single positive MEP waveform was recorded in the BF, TA, and FHL of both hind limbs.

To further corroborate these findings, MEP were assessed 2 months after the surgical intervention in both groups. In the experimental group, the MEP waveforms observed for the BF, TA, and FHL muscles in the lower limbs closely resembled those of the APL muscle in the upper limbs, indicating substantial recovery. In contrast, the control group exhibited less significant recovery in MEP waveforms of the lower limbs (Figure 5). These results underscore the successful restoration of motor function in the experimental group and highlight the positive impact of PEG and tacrolimus intervention.

### 3.3 Imaging observation of spinal cord graft and spinal cord neural continuity after vASCT

**FIGURE 6:**
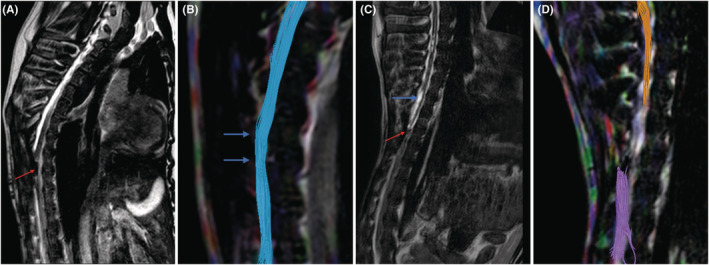
T2WI and DTI analysis of experimental and control groups at 2 months post‐surgery. At the 2‐month mark post‐surgery, T2‐weighted imaging (T2WI) and Diffusion Tensor Imaging (DTI) were employed to assess the outcomes in both the experimental and control groups. In the experimental group, T2WI displayed a spinal cord graft with a relatively normal appearance and signal intensity, indicating successful reconstruction of spinal cord continuity (arrow in A). DTI in the experimental group revealed fusion and connection of nerve fibers at the two contact surfaces of the spinal cord graft and the recipient spinal cord (arrows in B). Conversely, T2WI in the control group revealed a smaller graft size, indicative of necrosis (red arrow in C), and cystic vacuolization in the nearby recipient spinal cord tissue (blue arrow in C). DTI in the control group demonstrated disruption of spinal cord continuity and the presence of large nerve fiber defects (D).

In the experimental group, T2‐weighted images (T2WI) revealed spinal cord grafts with nearly normal signal intensity (Figure 6A), whereas the graft volume in the control group diminished, attributed to necrosis, accompanied by substantial cystic degeneration proximal to the graft site (Figure 6C). Furthermore, DTI in the experimental group showed restored spinal cord continuity with a seamless fusion between the graft and recipient spinal cords (Figure 6B). Conversely, the control group displayed disrupted spinal cord continuity, absence of nerve fiber imaging in the graft area, and significant nerve defects resulting from cystic degeneration in the proximal spinal cord (Figure 6D).

To provide a more quantitative assessment of spinal cord graft survival, we analyzed the fractional anisotropy (FA) and apparent diffusion coefficient (ADC) parameters. FA values indicate the degree of directionality in water molecule diffusion, with higher values signifying improved myelin sheath and nerve fiber bundle integrity, correlating with enhanced nerve fiber conduction. Conversely, ADC values represent the average diffusion ability of water molecules in all directions, with elevated ADC values indicating increased water molecule diffusion capacity, suggesting more severe structural damage to nerve fibers.^18^

**FIGURE 7:**
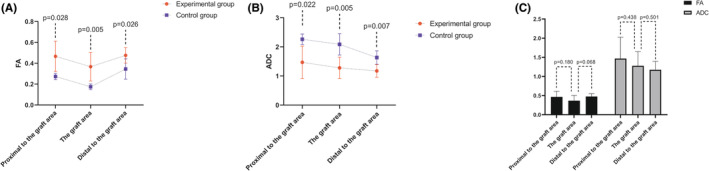
DTI‐related measures: FA and ADC. DTI‐related measures encompassing FA and ADC were assessed. The FA values of the transplanted spinal cord tissue, as well as the distal and proximal spinal cord (located one vertebra away from the graft area), exhibited a significant increase in the experimental group compared to the control group (*p* < 0.05, A). Conversely, the ADC values of the transplanted spinal cord tissue, as well as the distal and proximal spinal cord (located one vertebra away from the graft area), demonstrated a significant decrease in the experimental group compared to the control group (*p* < 0.05, B). Notably, the FA and ADC values of the transplanted spinal cord tissue did not exhibit significant differences from those of the distal and proximal spinal cord (located one vertebra away from the graft area) in the experimental group (*p* > 0.05, C).

In our study, the FA values for the spinal cord grafts and both the distal and proximal spinal cords in the experimental group were significantly higher compared to those in the control group (*p* < 0.05, Table 3, Figure 7A). Additionally, ADC values in the experimental group were notably lower than those in the control group (*p* < 0.05, Table 3, Figure 7B). Importantly, the FA and ADC values at the spinal cord graft site did not significantly differ from those at the distal and proximal spinal cord in the experimental group (*p* > 0.05, Figure 7C), consistent with the findings from MRI and DTI imaging.

**TABLE 3:** Comparative analysis of DTI‐related data metrics in Beagles between the experimental and control groups.

DTI parameter	Proximal to the graft area	The graft area	Distal to the graft area
FA (mean ± SD)			
Experimental group	0.467 ± 0.145	0.367 ± 0.137	0.476 ± 0.074
Control group	0.273 ± 0.028	0.175 ± 0.027	0.345 ± 0.097
*t* Value	2.578	3.825	2.620
*p* Value	0.028	0.005	0.026
ADC (mean ± SD)			
Experimental group	1.468 ± 0.558	1.279 ± 0.368	1.174 ± 0.221
Control group	2.261 ± 0.180	2.086 ± 0.365	1.634 ± 0.233
*t* Value	−2.712	−3.589	−3.345
*p* Value	0.022	0.005	0.007

Abbreviations: ADC, apparent diffusion coefficient; DTI, diffusion tensor imaging; FA, fractional anisotropy; SD, standard deviation.

## 4 DISCUSSION

In cases of spinal cord injuries resulting from traumatic factors, the spinal cord tissue undergoes two stages, encompassing both primary and secondary injury phases.^19^ The irreversibility of SCI is largely attributed to the persistence of the secondary injury processes. During the early stages of secondary injury, the affected region exhibits characteristics such as edema, hemorrhage, ischemia, inflammatory cell infiltration, release of cytotoxic substances, and cellular demise. Subsequent events involve nerve cell necrosis, demyelination, and structural damage, culminating in the formation of cystic microcavities. In response, astrocytes proliferate and deposit extracellular matrix molecules in the vicinity of the lesion site. These astrocytic scars mature into glial scars, which impede nerve regeneration, while the cystic cavities coalesce further hinder axon regeneration and cell migration.^20^ Drawing from imaging and histological data derived from our clinical trial, we observed that the SCI site in patients with traumatic paraplegia exhibited a substantial spinal cord defect. This defect was characterized by the infiltration of glial scar tissue and cystic cavities.^7^, ^8^ Much like how organs in kidney and heart failure, as well as end‐stage liver disease, lose their original functionality, a segment of tissue within the SCI site loses its innate capability for signal conduction. Following the treatment paradigm of organ transplantation and vCTA, a potential solution to the clinical challenges posed by SCI involves the removal of scar tissue from the injured area and the transplantation of a segment of normal functional spinal cord tissue.

vASCT, a novel surgical approach for SCF, has been developed by our team. The operation of vASCT involves the transection of spinal cord tissue with a scalpel, resulting in a SCI of this nature, which can potentially trigger a sequence of secondary injuries. Despite this, it remains a perplexing question as to why beagles subjected to vASCT can still exhibit a restoration of neurological function in their hind limbs.

To begin with, the surgical procedure involves the use of an extremely sharp knife, which exerts a force of 10 N to rapidly incise the spinal cord. This force is markedly lower when compared to the force of SCI resulting from events such as traffic accidents or falls from heights, which can reach as high as 26,000 N, representing a substantial difference of 2600 times. It's important to note that the primary SCI incurred during surgery is considerably less severe than the trauma‐induced injuries.^21^

Furthermore, traumatic spinal cord injuries lead to a disruption of the spinal cord's blood supply, resulting in severe ischemia during the secondary injury phase. In our vASCT procedure, the blood supply to the spinal cord graft is restored through vascular anastomosis of the vascular pedicle. This critical step serves to mitigate the influence of ischemia as a contributing factor in the secondary injury.

Moreover, our research team has extensively validated the efficacy of PEG in the treatment of SCI through an extensive body of work, including various animal models and clinical trials conducted over the years.^1^, ^2^, ^3^, ^4^, ^6^, ^7^, ^8^ In the previous study, PEG has also demonstrated the ability to shield neurons and glial cells from the detrimental effects of inflammation, cytotoxic substances, and oxidative stress during the secondary injury phase. Additionally, PEG exhibits a membrane‐protective effect by facilitating membrane resealing. This is achieved by reducing membrane surface tension and enhancing membrane fluidity, conditions conducive to acute membrane “resealing” in transected nerves.^22^ In vitro experiments have shown that PEG can fuse and repair axonal membranes within minutes, thus reinstating neural continuity and preserving action potential conductivity in severed axons.^23^, ^24^

In our current investigation, PEG was locally applied at the interface between the donor spinal cord graft and the recipient spinal cord stumps during vASCT. The effectiveness of PEG was substantiated through various metrics, including the recovery of SCEP positive waveforms (Figure 4C), motor function in the hind limbs (Figure 3), the restoration of lower limb MEP signal conduction (Figure 5), and the reestablishment of spinal cord neural continuity as demonstrated by DTI post‐surgery in the experimental animals (Figure 6B).

In the control group, issues emerged related to postoperative immune rejection. This resulted in the failure of fusion between the transplanted spinal cord graft and the recipient spinal cord, leading to necrosis in the spinal cord graft and surrounding tissue. Additionally, the cystic cavities were observed (Figure 6C). Beagles in the control group experienced persistent hind limb paralysis post‐surgery (Figure 3), with no recovery in electrophysiological MEP waveforms (Figure 5). Furthermore, DTI revealed a significant interruption in spinal cord continuity, characterized by a substantial segmental defect (Figure 6D).

Spinal cord ischemia, or inadequate blood flow, inflicts substantial damage on the spinal cord tissue. Histologically, gray matter ischemic injury presents as cytoplasmic eosinophilia with cytoarchitecture disintegration, nuclear pyknosis, cell body shrinkage, and vacuolation within the neuropil. In the white matter, widespread vacuolation is evident.^25^ Notably, during the initial 5 min of spinal cord ischemia, venules dilate due to red blood cell aggregation, though axonal changes remain absent. However, from 15 to 30 min, there is a progression involving a modest degree of hemorrhage, red blood cell extravasation, and axonal alterations. By 4 h, myelin destruction, axonal degeneration, and ischemic endothelial damage become prominent.^26^ It's essential to recognize that the duration of ischemia following spinal cord graft isolation has a direct correlation with its impact on the nerve tissue of the graft. Furthermore, restoring blood supply to ischemic tissue can lead to additional damage, known as reperfusion injury. A key mechanism in this process is the generation of reactive oxygen species (ROS) post‐reperfusion. Specifically, the surge in free radicals can lead to cell membrane and organelle damage, lipid peroxidation, inactivation of crucial enzyme systems, induction of neuronal apoptosis, and ultimately, severe impairment of spinal cord function.^27^ Neurons, with their limited antioxidant defenses, are particularly susceptible to ROS‐induced damage.^28^

Previous research has established that a critical ischemic time of 3 h is essential for the potential recovery of the spinal cord. Treating acute SCI solely through spinal cord revascularization, within a time frame of less than 4 h and without additional treatment, offers a promising avenue for neurological benefits.^26^ In our study, the sequence of surgical procedures was meticulously designed to prioritize spinal cord graft revascularization (Figure 1), ensuring that the mean spinal cord ischemia time was 2 h in the experimental group and 2 h and 11 min in the control group (Table 1). Furthermore, immediately after isolating the donor spinal cord graft, we employed hypothermia therapy by covering the spinal cord tissue with ice‐cold saline gauze. Hypothermia is a comprehensive neuroprotective treatment with multiple mechanisms of action in SCI. These mechanisms include the reduction of hemorrhage, edema, stress, and potential attenuation of glutamate excitotoxicity, oxidative stress, inflammation, and apoptosis.^29^

Tacrolimus, also known as FK506, is an increasingly prevalent immunosuppressant with global use. FDA‐approved for preventing allograft rejection, its mechanism of action involves the inhibition of interleukin‐2, calcineurin, and calmodulin‐dependent phosphatase.^30^, ^31^ Over the course of various clinical trials, tacrolimus has evolved into a fundamental component of modern immunosuppressive therapy for both organ transplantation and vCTA. In addition, tacrolimus has demonstrated neuroprotective and neuroregenerative properties, particularly within the nervous system. Studies conducted in models of cerebral ischemia have revealed that tacrolimus exerts neuroprotective effects by mitigating nitricoxidesynthase‐mediated neurotoxicity through the inhibition of calcineurin.^32^, ^33^ Research has clarified that the capacity of tacrolimus to alleviate ischemia–reperfusion injury arises from its comprehensive impact on microcirculation, free radical metabolism, calcium activation pathways, inflammatory cascades, mitochondrial stability, apoptosis, stress response proteins, and tissue recovery.^34^, ^35^ Furthermore, certain studies have indicated that both low and high doses of tacrolimus can influence nerve regeneration.^36^ In our study, the experimental group received a low‐dose tacrolimus and methylprednisolone immunosuppressive regimen. Consequently, we cannot discount the possibility that the favorable outcomes, including the successful survival of spinal cord grafts, the restoration of spinal cord continuity, and the recovery of hind limb motor function, may be linked to tacrolimus's impact on nerve regeneration and its ability to mitigate ischemia–reperfusion effects. The effectiveness of low‐dose tacrolimus in preventing immune rejection in the context of vASCT was further substantiated by the imaging evidence of the positive survival outcomes in beagles from the experimental group (Figure 6A,B).

## 5 CONCLUSION

This study presents the pioneering evidence that the use of vASCT can facilitate the restoration of spinal cord neural continuity and hindlimb motor function in a beagle model with a spinal cord defect. Notably, the combination of PEG and tacrolimus has emerged as a crucial factor in the reconstruction of spinal cord continuity and the restoration of hind limb motor function within this context. Moreover, tacrolimus has demonstrated commendable anti‐immune rejection efficacy in the vASCT model involving beagles. It is important to underscore that while the findings of this study present an initial demonstration of the potential efficacy of vASCT as a prospective treatment for patients with SCI, continued comprehensive research remains essential to refine and substantiate its clinical applicability.

## CONFLICT OF INTEREST STATEMENT

The authors of this manuscript have no conflicts of interest to disclose.

## Supporting information


Video S1

## Data Availability

The data that support the findings of this study are available from the corresponding author upon reasonable request.

## References

[1] Ye Y , Kim CY , Miao Q , Ren X . Fusogen‐assisted rapid reconstitution of anatomophysiologic continuity of the transected spinal cord. Surgery. 2016;160(1):20‐25. 27138179 10.1016/j.surg.2016.03.023

[2] Kim CY , Oh H , Ren X , Canavero S . Immunohistochemical evidence of axonal regrowth across polyethylene glycol‐fused cervical cords in mice. Neural Regen Res. 2017;12(1):149‐150. 28250761 10.4103/1673-5374.199014 PMC5319221

[3] Ren S , Liu ZH , Wu Q , et al. Polyethylene glycol‐induced motor recovery after total spinal transection in rats. CNS Neurosci Ther. 2017;23(8):680‐685. 28612398 10.1111/cns.12713 PMC6492641

[4] Liu Z , Ren S , Fu K , et al. Restoration of motor function after operative reconstruction of the acutely transected spinal cord in the canine model. Surgery. 2018;163(5):976‐983. 29223327 10.1016/j.surg.2017.10.015

[5] Ren X , Canavero S (Eds.). The technology of head transplantation. New Developments in Medical Research. Nova Science Publishers; 2020.

[6] Ren S , Zhang W , Liu H , et al. Transplantation of a vascularized pedicle of hemisected spinal cord to establish spinal cord continuity after removal of a segment of the thoracic spinal cord: a proof‐of‐principle study in dogs. CNS Neurosci Ther. 2021;27:1182‐1197. 34184402 10.1111/cns.13696 PMC8446222

[7] Ren X , Zhang W , Qin J , et al. Partial restoration of spinal cord neural continuity via vascular pedicle hemisected spinal cord transplantation using spinal cord fusion technique. CNS Neurosci Ther. 2022;28:1205‐1217. 35545932 10.1111/cns.13853 PMC9253790

[8] Ren X , Zhang W , Mo J , et al. Partial restoration of spinal cord neural continuity via sural nerve transplantation using a technique of spinal cord fusion. Front Neurosci. 2022;16:808983. 35237120 10.3389/fnins.2022.808983 PMC8882688

[9] Beyar R . Challenges in organ transplantation. Rambam Maimonides Med J. 2011;2(2):e0049. 23908807 10.5041/RMMJ.10049 PMC3678939

[10] Jones J , Gruber S , Barker J , Breidenbach W . Successful hand transplantation. One‐year follow‐up. Louisville Hand Transplant Team. N Engl J Med. 2000;343(7):468‐473. 10950668 10.1056/NEJM200008173430704

[11] Francois C , Breidenbach W , Maldonado C , et al. Hand transplantation: comparisons and observations of the first four clinical cases. Microsurgery. 2000;20(8):360‐371. 11150985 10.1002/1098-2752(2000)20:8<360::aid-micr4>3.0.co;2-e

[12] Shen H , Chen X , Li X , Jia K , Xiao Z , Dai J . Transplantation of adult spinal cord grafts into spinal cord transected rats improves their locomotor function. Sci China Life Sci. 2019;62(6):725‐733. 30915628 10.1007/s11427-019-9490-8

[13] Shen H , Wu S , Chen X , et al. Allotransplantation of adult spinal cord tissues after complete transected spinal cord injury: long‐term survival and functional recovery in canines. Sci China Life Sci. 2020;63(12):1879‐1886. 32382980 10.1007/s11427-019-1623-5

[14] Boutron I , Percie du Sert N , Ahluwalia A , et al. Reporting animal research: explanation and elaboration for the ARRIVE guidelines 2.0. PLoS Biol. 2020;18(7):e3000411. 32663221 10.1371/journal.pbio.3000411 PMC7360025

[15] Song RB , Basso DM , da Costa RC , Fisher LC , Mo X , Moore SA . Adaptation of the Basso‐Beattie‐Bresnahan locomotor rating scale for use in a clinical model of spinal cord injury in dogs. J Neurosci Methods. 2016;268:117‐124. 27155106 10.1016/j.jneumeth.2016.04.023 PMC4903932

[16] Hendrix P , Griessenauer CJ , Cohen‐Adad J , et al. Spinal diffusion tensor imaging: a comprehensive review with emphasis on spinal cord anatomy and clinical applications. Clin Anat. 2015;28(1):88‐95. 24497009 10.1002/ca.22349

[17] D'Souza MM , Choudhary A , Poonia M , Kumar P , Khushu S . Diffusion tensor MR imaging in spinal cord injury. Injury. 2017;48(4):880‐884. 28242068 10.1016/j.injury.2017.02.016

[18] Zhao Y , Yao L , Ao L , Ou J , He Y , Shang Y . Study of the diffusion tensor imaging for preclinical therapeutic efficacy of umbilical cord mesenchymal stem cell transplantation in the treatment of spinal cord injury. Int J Gen Med. 2021;14:9721‐9732. 34938101 10.2147/IJGM.S326023 PMC8686231

[19] Hilton BJ , Moulson AJ , Tetzlaff W . Neuroprotection and secondary damage following spinal cord injury: concepts and methods. Neurosci Lett. 2017;652:3‐10. 27939975 10.1016/j.neulet.2016.12.004

[20] Ahuja CS , Wilson JR , Nori S , et al. Traumatic spinal cord injury. Nat Rev Dis Primers. 2017;3:17018. 28447605 10.1038/nrdp.2017.18

[21] Sledge J , Graham WA , Westmoreland S , et al. Spinal cord injury models in non human primates: are lesions created by sharp instruments relevant to human injuries? Med Hypotheses. 2013;81(4):747‐748. 23948598 10.1016/j.mehy.2013.07.040

[22] Shi R . Polyethylene glycol repairs membrane damage and enhances functional recovery: a tissue engineering approach to spinal cord injury. Neurosci Bull. 2013;29(4):460‐466. 23893430 10.1007/s12264-013-1364-5 PMC5561946

[23] Bittner G , Ballinger M , Raymond M . Reconnection of severed nerve axons with polyethylene glycol. Brain Res. 1986;367:351‐355. 3697710 10.1016/0006-8993(86)91617-3

[24] Shi R , Borgens R , Blight A . Functional reconnection of severed mammalian spinal cord axons with polyethylene glycol. J Neurotrauma. 1999;16(8):727‐738. 10511246 10.1089/neu.1999.16.727

[25] Kanellopoulos G , Xu X , Hsu C , Lu X , Sundt T , Kouchoukos N . White matter injury in spinal cord ischemia: protection by AMPA/kainate glutamate receptor antagonism. Stroke. 2000;31(8):1945‐1952. 10926962 10.1161/01.str.31.8.1945

[26] Bitar Alatorre WE , Garcia Martinez D , Rosales Corral SA , Flores Soto ME , Velarde Silva G , Portilla de Buen E . Critical ischemia time in a model of spinal cord section. A study performed on dogs. Eur Spine J. 2006;16(4):563‐572. 17024402 10.1007/s00586-006-0222-9 PMC2229824

[27] Zweier J , Talukder M . The role of oxidants and free radicals in reperfusion injury. Cardiovasc Res. 2006;70(2):181‐190. 16580655 10.1016/j.cardiores.2006.02.025

[28] Li Z , Zhang B , Yao W , Zhang C , Wan L , Zhang Y . APC‐Cdh1 regulates neuronal apoptosis through modulating glycolysis and pentose‐phosphate pathway after oxygen‐glucose deprivation and reperfusion. Cell Mol Neurobiol. 2018;39(1):123‐135. 30460429 10.1007/s10571-018-0638-x PMC11469847

[29] Kline AE , Batchelor PE , Skeers P , et al. Systematic review and meta‐analysis of therapeutic hypothermia in animal models of spinal cord injury. PLoS One. 2013;8(8):e71317. 23951131 10.1371/journal.pone.0071317 PMC3739756

[30] Tung TH . Tacrolimus (FK506): safety and applications in reconstructive surgery. Hand. 2009;5(1):1‐8. 19363638 10.1007/s11552-009-9193-8 PMC2820618

[31] Pamuk F , Cetinkaya BO , Ayas B , Keles GC , Gacar A . Evaluation of gingival alterations in rats medicated with cyclosporine a, tacrolimus and sirolimus: a stereological study. J Periodontal Res. 2014;50(5):629‐636. 25399832 10.1111/jre.12243

[32] Dawson T , Steiner J , Dawson V , Dinerman J , Uhl G , Snyder S . Immunosuppressant FK506 enhances phosphorylation of nitric oxide synthase and protects against glutamate neurotoxicity. Proc Natl Acad Sci USA. 1993;90(21):9808‐9812. 7694293 10.1073/pnas.90.21.9808 PMC47661

[33] Sobrado M , Ramirez B , Neria F , et al. Regulator of calcineurin 1 (Rcan1) has a protective role in brain ischemia/reperfusion injury. J Neuroinflammation. 2012;9:48. 22397398 10.1186/1742-2094-9-48 PMC3325863

[34] St Peter SD , Moss AA , Mulligan DC . Effects of tacrolimus on ischemia‐reperfusion injury. Liver Transpl. 2003;9(2):105‐116. 12548502 10.1053/jlts.2003.50020

[35] Micó‐Carnero M , Zaouali MA , Rojano‐Alfonso C , Maroto‐Serrat C , Ben Abdennebi H , Peralta C . A potential route to reduce ischemia/reperfusion injury in organ preservation. Cells. 2022;11(17):2763. 36078175 10.3390/cells11172763 PMC9455584

[36] Udina E , Ceballos D , Verdú E , Gold BG , Navarro X . Bimodal dose‐dependence of FK506 on the rate of axonal regeneration in mouse peripheral nerve. Muscle Nerve. 2002;26(3):348‐355. 12210363 10.1002/mus.10195

